# Secondary placement of adjustable continence therapy (ProACT™) using open perineal technique: Case report of ProACT placement in a man with a devastated urethra following pelvic trauma and multiple AUS erosions

**DOI:** 10.1016/j.eucr.2023.102424

**Published:** 2023-05-24

**Authors:** Lily Kong, Nathaniel D. Coddington, Brian J. Flynn

**Affiliations:** University of Colorado Anschutz Medical Campus, 13001, E 17th Pl., Aurora, CO, 80045, USA

**Keywords:** ProACT, Male stress urinary incontinence, Devastated urethra

## Abstract

Adjustable continence therapy (ProACT) is an underutilized treatment option in men with stress urinary incontinence. The device is placed using a perineal percutaneous tunneled approach. We demonstrate a salvage technique for ProACT placement in a man with a devastated urethra following pelvic trauma and multiple artificial urinary sphincter (AUS) erosions who failed a tunneled approach. Our novel technique has utility in those at high risk for intra-operative trocar injury to the urinary tract with a tunneled approach. An open approach may also be a viable option in high-risk patients who have failed a conventional ProACT approach, male sling, or AUS.

## 1 Introduction

Male stress urinary incontinence (SUI) can occur following therapy for prostate cancer therapy, with up to 40% of individuals experiencing post-prostatectomy incontinence.^1^ SUI may also be caused by sphincter dysfunction related to neurologic conditions or pelvic trauma.^2^ The artificial urinary sphincter (AUS) was the first commercially available treatment for male SUI and remains the gold standard for moderate to severe incontinence in men.^3^ SUI treatment options have gradually expanded to include passive devices such as the urethral sling and other compressive devices. The adjustable continence therapy (ProACT™) system is a minimally invasive treatment for mild, moderate, and severe SUI that can be adjusted percutaneously and is a passive device in that, unlike the AUS, it requires no active patient manipulation. The ProACT system consists of two silicone balloons placed on each side of the urethra at the level of the bladder neck using a special trocar tunneled across the perineum. Each balloon is attached to an adjustment port which is placed in the inferior scrotum for percutaneous access.^4^ We describe placement of ProACT device using an open perineal approach in a patient with a devastated urethra due to pelvic trauma and history of multiple AUS erosion who previously failed a tunneled approach.^5^

## 2 Case presentation

**Fig. 1:**
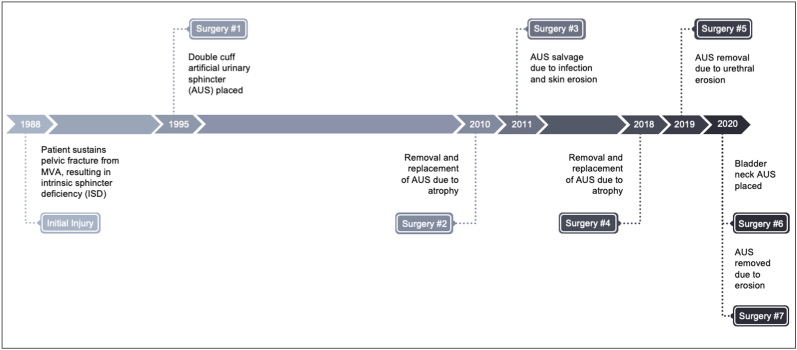
AUS surgery timeline detailing multiple surgeries for incontinence, atrophy, and erosion.

Our patient is a 53-year-old male with a history of intrinsic sphincter deficiency (ISD) following pelvic fracture from a motor vehicle accident. He has a past medical history of cirrhosis, thrombocytopenia, and lymphedema making him a poor candidate for urinary diversion. He had a bulbar AUS placed 7 years after his injury and had a good outcome for 25 years until he developed recurrent incontinence due to urethral atrophy. The device was replaced, and the cuff downsized, however the scrotal tubing eroded through the skin a year later, requiring device salvage. Subsequently, he had multiple AUS revisions for atrophy and erosion resulting in total incontinence. A detailed AUS surgery timeline is provided below (Fig. 1).

**Fig. 2:**
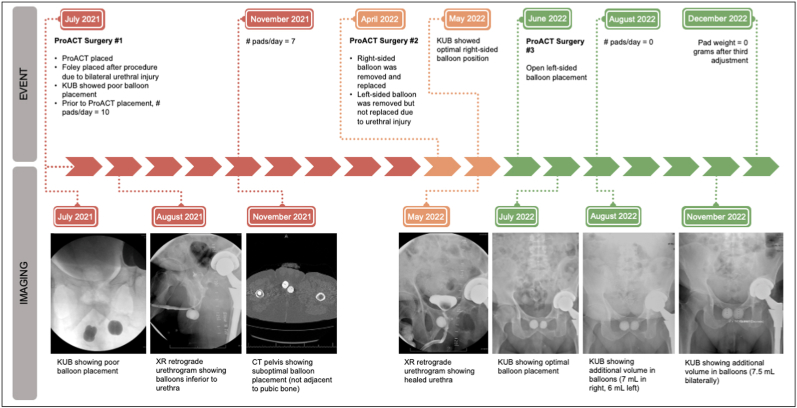
ProACT surgery timeline detailing surgical history with associated imaging.

After multiple erosions and a discussion of remaining treatment options, the patient elected to undergo ProACT using the standard tunneled technique.^4^ A detailed ProACT surgery timeline is provided below (Fig. 2). Post-operatively, the patient had minimal improvement, and the balloons were noted to be in a suboptimal position inferior to the urethra (Fig. 3a, Fig. 3b). The patient then had a bilateral revision in the standard fashion. During the revision, the right-sided balloon was successfully removed and replaced; the left-sided balloon was removed, but a new balloon could not be successfully placed due to urethral injury from the trocar, which prohibited safe placement. Subsequent retrograde urethrogram showed no urethral extravasation and optimal balloon position on the right side. Given the patient's limited options and failed percutaneous approach on the left, a decision was made to proceed with an open perineal placement to achieve safe and optimal positioning of the left-sided balloon.

**Fig. 3a:**
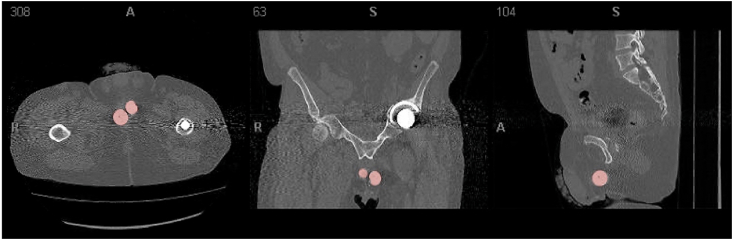
CT pelvis demonstrating suboptimal balloon position (red) position after the initial ProACT™ surgery. (For interpretation of the references to colour in this figure legend, the reader is referred to the Web version of this article.)

**Fig. 3b:**
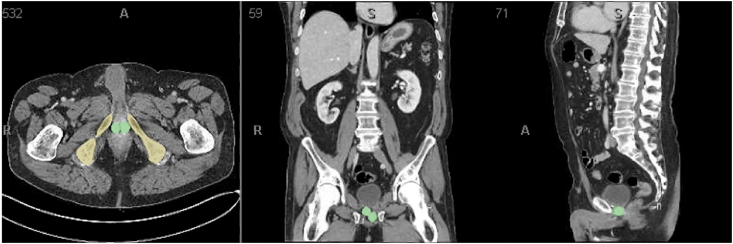
CT pelvis demonstrating optimal balloon position (green) adjacent to the pubic bone (yellow) in a different patient. (For interpretation of the references to colour in this figure legend, the reader is referred to the Web version of this article.)

During the third ProACT surgery, intra-operative cystourethroscopy demonstrated significant scarring of the bulbar and prostatic urethra limiting the tunneled approach. Therefore, a midline perineal incision was made, and the left side of the urethra was dissected. Significant fibrosis was encountered, and the bulbar urethra was found to be scarred to the inferior pubic rami, likely explaining the prior difficulty with balloon placement. A curved Cobb periosteal elevator was used to develop a space adjacent to the left ischial pubic ramus. Implantation then proceeded in the standard fashion. A sharp trocar mated with a U-channel sheath was directed toward the bladder neck under fluoroscopy, with the cystoscope as a landmark at the bladder neck. The sharp trocar was replaced with a blunt trocar via the sheath to dilate the last 0.5 cm to reduce the risk of perforation of the proximal urethra or bladder neck. The blunt trocar was removed and the balloon advanced into position via the U-channel sheath under fluoroscopic guidance. The left-sided balloon was inflated with 2 mL of isotonic contrast solution. The position of the balloon relative to the urethra was monitored via simultaneous fluoroscopy and cystoscopy, which demonstrated good coaptation. The pipette was tunneled into the left hemiscrotum, and the incision closed in standard fashion. The patient tolerated the procedure well with no complications and or blood loss.

One month post-operatively, the patient reported improved continence. Following three ProACT adjustments, he had complete resolution of his incontinence, as evidenced by a 24-h pad weight of zero grams. Post-operative imaging demonstrated excellent balloon placement with no evidence of migration.

## 3 Discussion

Safe AUS placement may not be feasible in all patients due to a compromised urethra from prior urethroplasty, erosion or radiation. Moreover, not all patients have the physical skills or te cognitive skills to operate, an active device. Our patient had a history of severe injury to his posterior urethra, further complicating traditional first-line approaches. We demonstrated that ProACT is a viable option in patients who have failed or are not candidates for traditional continence mechanisms, such as male perineal sling and AUS.^5^ Given the patient's devastated posterior urethra, we explored a novel open technique for placing the device that allowed us to achieve appropriate positioning of the balloons. Peri-urethral scarring, typically due to radiation therapy, prior surgery and/or urethral erosion can lead to an inferior result when compared to cases without these factors. However, this case report demonstrated that despite adverse implantation features success can still be achieved.^4^ The likelihood of erosion has been variable reported in European series, but under 5% in the pooled analysis.^4^ However, these are overwhelming secondary to prostate therapy (prostatectomy, radiation, TURP). There is no reported use of ProACT in patients with pelvic trauma. To our knowledge, this is the first report in the literature of an open approach for adjustable continence therapy (ProACT).

## 4 Conclusion

We present a successful alternative surgical treatment option for severe male SUI with the ProACT device using an open perineal approach in a patient with devastated urethra. ProACT may be considered as a second-line option in patients who have failed or are not candidates for traditional continence therapies.

## Consent

Informed consent was obtained from the patient for publication of this case report and accompanying images.

## Funding

This research received no specific grant from funding agencies from the public, commercial, or not-for-profit sectors.

## Declaration of competing interest

Brian J. Flynn, MD is an investigator for Cook Myosite, Boston Scientific and Uromedica.
